# Reply to Watt

**DOI:** 10.1093/cid/ciz1192

**Published:** 2019-12-14

**Authors:** Tri Wangrangsimakul, Weerawat Phuklia, Paul N Newton, Allen L Richards, Nicholas P J Day

**Affiliations:** 1 Mahidol-Oxford Tropical Medicine Research Unit, Faculty of Tropical Medicine, Mahidol University, Bangkok, Thailand; 2 Centre for Tropical Medicine and Global Health, Nuffield Department of Clinical Medicine, University of Oxford, Oxford, United Kingdom; 3 Lao-Oxford-Mahosot Hospital–Wellcome Trust Research Unit, Microbiology Laboratory, Mahosot Hospital, Vientiane, Lao People’s Democratic Republic; 4 Department of Preventive Medicine and Biostatistics, Uniformed Services University of the Health Sciences, Bethesda, Maryland, USA

To the Editor—We thank the author for his letter [1]. At the heart of the debate is the issue of how antibiotic resistance for *Orientia* species should be defined. We applied strictly microbiological criteria to doxycycline resistance, based on minimum inhibitory concentrations (MICs) and the pharmacokinetic/pharmacodynamic (PK/PD) properties of the tested antibiotics [2]. Doxycycline MICs obtained for AFC-3 and AFSC-4 *Orientia tsutsugamushi* isolates were recently reported at 0.125 mg/L and 0.250 mg/L, respectively [3]. These values fall within the expected wild-type distribution of doxycycline MICs (median of 0.125 mg/L ± one to two 2-fold dilution steps) in 5 reference strains (including Karp and Gilliam, prototypical strains considered doxycycline susceptible) and 51 clinical isolates from Laos and Thailand, and remain well below the plasma concentrations achieved in humans at standard doses (100–200 mg/day, peak serum concentrations of 1.7–5.9 mg/L) [3–5]. The small differences between doxycycline MICs observed are likely due to technical variation rather than major differences in susceptibility.

The author stipulates that attenuated clinical response to doxycycline implies diminished susceptibility and dismisses other important factors known to influence infection such as bacterial virulence and host immunity [1]. We do not dispute that some scrub typhus patients in Chiangrai have prolonged fever clearance times despite appropriate antibiotic treatment. However, fever clearance times ≥72 hours were observed in some patients receiving doxycycline, rifampicin, azithromycin, and chloramphenicol as part of clinical trials conducted in Thailand and in South Korea [6–10].

Results of the mouse survivability assay may be influenced by virulence of the infecting *O. tsutsugamushi* isolates [11]. The differences in pharmacokinetics of antibiotics in mice and lack of comparison to other reference and clinical isolates from patients with appropriate responses to treatment are additional weaknesses [12, 13]. The author’s work shows that mouse survivability rates were similar for doxycycline and azithromycin when infected with Chiangrai *O. tsutsugamushi* isolates [14]. If diminished antibiotic susceptibility is determined by attenuated clinical response and reduced mouse survivability, then resistance in scrub typhus to the main antibiotics used appear widespread. Current evidence suggests that other factors described in our article influence clinical response and that a microbiological basis for doxycycline resistance in *O. tsutsugamushi* is lacking.

Chiangrai patients from the author’s original study were on average febrile for longer prior to admission than patients from Mae Sot [13]. Although statistical significance was not reached, the differences may have potentially affected outcome, and larger studies to assess the relationship between age and duration of fever to outcome are required. We referenced the author’s work while discussing potential sources of antibiotic selective pressure. There is evidence for the widespread use of antibiotics in animal feed globally and in Thailand, but we agree that detailed comparative studies at the provincial level are unavailable [15–18].

We believe that antibiotic susceptibility for *O. tsutsugamushi* should be based on MICs and PK/PD data and advocate the use of antimicrobial susceptibility tests that are accurate and reproducible, allowing for standardization and harmonization of results. Current trials embedding analysis of these important aspects should provide further clarity.

## Notes

***Financial support.*** T. W., W. P., P. N. N., and N. P. J. D. are funded by the Wellcome Trust, United Kingdom, as part of the Mahidol-Oxford Tropical Medicine Research Unit Tropical Health Network institutional funding support.

***Potential conflicts of interest.*** The authors: No reported conflicts of interest. All authors have submitted the ICMJE Form for Disclosure of Potential Conflicts of Interest.
